# Genetic Diversity of Wild and Cultivated Muscadine Grapes (*Vitis rotundifolia* Michx.)

**DOI:** 10.3389/fpls.2022.852130

**Published:** 2022-03-28

**Authors:** Kenneth Buck, Margaret Worthington

**Affiliations:** Department of Horticulture, University of Arkansas, Fayetteville, AR, United States

**Keywords:** *Muscadinia rotundifolia*, *Vitis munsoniana*, genetic diversity, population structure, phylogeny

## Abstract

The muscadine (*Vitis rotundifolia* syn. *Muscadinia rotundifolia*) is an American grape species native to the southeastern United States that has been cultivated for centuries. Muscadines are one of three grape species in subgenus *Muscadinia* with a chromosome number of 2*n* = 40 (*V. rotundifolia*, *Vitis munsoniana*, and *Vitis popenoei*), making them genetically distinct from the European wine and table grape (*Vitis vinifera*) and other species in subgenus *Euvitis*. Crop improvement efforts have been continuous since the late 19th century, yet the germplasm that served as the foundation for early muscadine breeding efforts was sourced from a relatively small portion of their native range, mostly in the coastal plains of North Carolina. This study used the rhAmpSeq *Vitis* core panel haplotype markers to genotype 194 *Muscadinia* accessions from five cultivated populations and 15 wild populations collected across their native range. Wild populations from the western half of the native range were generally less genetically differentiated than hypothesized, but were genetically distinct from the material used in both past and present breeding efforts. One population collected from coastal North Carolina grouped closely with *V. munsoniana* accessions despite being well outside the reported range for that species. Principal coordinate and *structure* analyses revealed three main groups within the 194 accessions: one for cultivated material, one for wild *V. rotundifolia*, and one for *V. munsoniana* and *V. popenoei*. At *K* = 5, *structure* results showed that more recent muscadine cultivars are further differentiated from wild accessions and varieties. These analyses confirmed our hypothesis that muscadine cultivars are genetically differentiated from their wild counterparts. This study also showed that genetic diversity in *V. rotundifolia* is not equally distributed across its native range and that the limited number of genotypes used in crop improvement efforts has not fully utilized the genetic diversity within the species.

## Introduction

The muscadine (*Vitis rotundifolia* Michx. syn. *Muscadinia rotundifolia* Simpson ex Munson) is a member of the grape family (Vitaceae) native to the southeastern United States. For centuries, this species has been used for both wine and fresh market production. The native range of the muscadine is from Maryland west to Texas and south to the Gulf Coast. Perhaps, the most important factor limiting the range of the species is cold hardiness. Although there are documented instances of muscadine vines surviving temperatures of −23°C, regions that regularly attain winter lows of −18°C are considered unsuitable for commercial muscadine production (Munson, 1909; Dearing, 1947; Clark, 2001).

Taxonomically, the genus *Vitis* can be split into two subgenera based on differences in chromosome number: *Euvitis* or bunch grapes (2*n* = 38) and *Muscadinia* (2*n* = 40). Major synteny between the *Euvitis* and *Muscadinia* genomes has been observed despite the difference in chromosome number and barriers to hybridization. Collinearity between chromosomes 7 and 20 in muscadines and chromosome 7 in bunch grapes suggests a chromosome fusion in *Euvitis* grapes (Blanc et al., 2012; Lewter et al., 2019; Cochetel et al., 2021). In addition to significant genetic differences, there are multiple phenotypic distinctions between muscadine grapes and the more familiar species in *Euvitis* such as the European wine and table grape (*Vitis vinifera* L.). Muscadines are resistant to many pathogens, such as Pierce’s Disease (*Xylella fastidiosa* Wells et al.), grapevine downy mildew [*Plasmopara viticola* (Berk. and M.A. Curtis) Berl. and De Toni], and powdery mildew [*Erysiphe necator* syn. *Uncinula necator* (Schw. Burr)] that make *V. vinifera* cultivation in the southeastern U.S. difficult (Hopkins et al., 1974; Merdinoglu et al., 2003; Ruel and Walker, 2006; Riaz et al., 2011). Muscadine flowers emerge 2–3 weeks later than *Euvitis* species in the same location and require approximately 100 days for the fruit to reach maturity (Goldy, 1992; Olien, 2001). Fruit size in muscadines is relatively large, often exceeding 2.5 cm in diameter, and fruit appears in clusters of 5–10 berries (Olien, 1990; Anderson, 2006; Conner, 2009). By contrast, *V. vinifera* fruit are smaller and appear in much larger clusters. Other distinguishing morphological differences in *Muscadinia* are unbranched tendrils, continuous pith through the nodes, grape abscission at maturity, and smooth bark (Comeaux et al., 1987; Olien, 1990).

Muscadines are the only economically important member of *Muscadinia*, although the other two species (*V. munsoniana* Simpson ex Munson and *V. popenoei* J. L. Fennell) have been used in some breeding efforts (Goldy and Onokpise, 2001). *Vitis munsoniana*, sometimes classified as a subspecies of *V. rotundifolia*, is endemic from southern Florida along the Gulf Coast to Texas and has not been reported in the interior regions of the South (Munson, 1909; Dearing, 1947). *Vitis popenoei* is a tropical grape species native to Central America and first described in southern Mexico (Fennell, 1940). It has been used sparingly in breeding efforts, most importantly in the pedigree of the cultivar “Southern Home” (Mortensen et al., 1994). The USDA National Clonal Germplasm Repository (NCGR) maintains a single accession of *V. popenoei* (DVIT 2970) collected from Veracruz, Mexico, affording the species a minor presence in previous molecular studies (e.g., Wan et al., 2013; Cao et al., 2020).

Muscadines have likely been cultivated by European colonists in the Americas since at least the 16th century, and by indigenous peoples for far longer (Bartram, 1791). Colonists in northern Florida were producing wine in the 1560s from large native grapes that were likely muscadines (Winsor, 2016). The wild variety “Scuppernong,” selected for its unique bronze fruit, has been cultivated for centuries and served as the foundation of the muscadine wine industry even into the early 20th century (Reimer and Detjen, 1910; Morton, 1988). Despite the long and well-recorded history of use in the southeastern U.S., the commercial muscadine industry did not begin until 1835 in North Carolina (Morton, 1988). By the early 20th century, one of the most popular wines in the United States was made from muscadines until Prohibition severely hampered the muscadine industry (Gohdes, 1982). By 1990, production had dropped to approximately 1,600 ha across the entire southeastern U.S. (Olien, 1990). In Arkansas, commercial muscadine vineyards were not present in the state until the mid-1970s (Moore, 1972; Clark, 2001), and the most recent literature estimates that muscadine production only accounts for approximately 3% of the total grape acreage in the state (Olien, 1990).

The earliest breeding efforts for muscadines began in the second half of the 19th century, with efforts to improve on the dominant variety “Scuppernong” and attempts at hybridizing muscadines and *V. vinifera* (Van Buren, 1871; Munson, 1909). Improvement from these private, individual-led programs were limited as hybridization efforts between the two subgenera were largely unsuccessful. Varieties originating from wild selections still constituted the majority of cultivated vines into the early 20th century (Husmann and Dearing, 1913). The USDA and North Carolina State University cooperatively founded the first public muscadine breeding program in 1908. Significant advances were made in this program, notably the development of perfect flowered muscadine cultivars (Dearing, 1917). Another breeding program at the University of Georgia (UGA) began in 1909 and has made significant strides in increasing vine yield, berry size, and fresh market berry quality over the last century (Conner, 2009). Currently, there are muscadine breeding efforts at the University of Arkansas System Division of Agriculture (UA), Florida A&M University, UGA, “Gardens Alive!” LLC, and the USDA-ARS Southern Horticultural Research Station in Poplarville, MS.

As the epicenter of muscadine culture and the location of the first public muscadine breeding program, eastern North Carolina was the main source of germplasm for early breeders (Husmann and Dearing, 1913). Wild vines from central Florida were also used in the very early stages of crop improvement. Before the establishment of the USDA cooperative vineyard in North Carolina, crosses were made between cultivated pistillate vines from North Carolina and wild pollinizers at a private vineyard in New Smyrna, FL (Dearing, 1917, 1947). The UGA breeding program only used three female varieties, “Flowers,” “Scuppernong,” and “Thomas,” to begin the program, and these were selected from varieties already in use by the North Carolina program (Stuckey, 1919). T.V. Munson, whose grape breeding efforts were based in east Texas, reported spending significant time canvassing the surrounding countryside in Texas and Oklahoma for exceptional wild grapes to use in his breeding efforts. Yet, he never mentions incorporating any wild muscadine vines from the region in his breeding efforts (Munson, 1909). In fact, there does not appear to be any record of wild accessions from west of the Appalachian Mountains used in any breeding program.

Multiple studies have utilized subgenus *Muscadinia* germplasm in phylogenetic research, although they often either represent a small percentage of the accessions analyzed or are specifically used as an outgroup for *Euvitis*-specific research (Zecca et al., 2012; Aradhya et al., 2013; Wan et al., 2013). Muscadine-specific conclusions that can be drawn from these studies are limited. There are relatively few studies specifically investigating the genetic diversity of muscadines. One comparative study found that bunch grapes had significantly more genetic variation than muscadines, although those results are difficult to interpret due to the low number of accessions compared and the interspecific background of the bunch grape accessions included in the study (Qu et al., 1996). Past studies using simple sequence repeat (SSR) markers to quantify genetic diversity in cultivated muscadines have found that observed heterozygosity was higher than expected (Riaz et al., 2008; Cao et al., 2020). However, pedigree analysis has found that recently released varieties have higher inbreeding levels than historical varieties (Williams et al., 2021). Marker data also show that allelic richness appears to decline between historical cultivars and cultivars released after 1970 (Cao et al., 2020). Despite the limited genetic base available to muscadine breeders and a loss of genetic diversity over time, inbreeding depression does not yet appear to be a significant issue in cultivated muscadines (Goldy and Onokpise, 2001), although low vine vigor has been cited as a potential effect of inbreeding already present in some breeding material (Williams et al., 2021).

Past research showed wild muscadine populations are sources of significant genetic variation. In one study, just nine wild muscadines accessions contained as many SSR alleles as 67 cultivated accessions (Cao et al., 2020). However, there is evidence to suggest that not every region harbors equal levels of genetic diversity. Smith (2010) conducted the only genetic research on *de novo* collected wild muscadine populations and found a diversity gradient running north–south, from more diverse populations in Florida to less diverse populations in North Carolina. Wild muscadine populations generally also appear to have lower observed heterozygosity than expected, although this did not result in a lack of genetic diversity as measured by the total number of alleles present compared to cultivated populations (Smith, 2010; Cao et al., 2020). Wild muscadines are potentially important sources of phenotypic variation for horticulturally important traits. In muscadines, bronze-colored grapes are preferred for winemaking because they lack the diglucoside anthocyanins that are highly susceptible to browning, which predominate in black-fruited cultivars (Robinson et al., 1966). Monoglucoside anthocyanins were thought to not exist in black muscadine fruit until they were detected by high-performance liquid chromatography (HPLC) in wild muscadine fruit samples collected from North Carolina and Arkansas (Goldy et al., 1989). Variation in resistance to Pierce’s Disease was observed in wild and cultivated muscadine accessions (Ruel and Walker, 2006). Although all muscadine vines used in that study were determined to be tolerant, differences in bacterial concentrations collected from various accessions indicated quantitative differences in resistance that may be important as pathogen pressures shift over time.

To date, no muscadine genetic diversity study has included a significant number of accessions from outside Florida or North Carolina. Considering that these states are also where wild vines were collected for use early breeding efforts, it is possible that a significant amount of genetic diversity is present in wild muscadine populations from unsampled regions, particularly the western half of its native range. This research investigates both the population structure and genetic diversity of cultivated populations and wild populations collected *de novo* from previously unsampled locations in the southeastern U.S.

## Materials and Methods

### Wild Population Sampling

Wild populations of *V. rotundifolia* and *V. munsoniana* were sampled from 15 sites in Alabama, Arkansas, Florida, Mississippi, North Carolina, Oklahoma, Tennessee, and Texas (Table 1). When multiple populations were collected within a state, populations were labeled from east to west with increasing numbers. These sampling locations included areas that had appeared in a previous phylogenetic study (Smith, 2010); regions that served as major germplasm sources for early breeding efforts, and areas in the western range of muscadines that have received little scientific attention. In addition, the collection sites represented a wide variety of environments to which muscadines are adapted (e.g., Ozark uplands and palm hammocks). At least 10 individual vines within a 1 km^2^ area were sampled from each collection site with a minimum of 5 m between individuals. After data filtration, the FLA2 population had only four individuals and was combined with the FLA population for analysis due to the proximity of the collection sites (25.1 km, Table 1). In addition to sampling 15 wild populations, a further four wild vines from the Ozark highlands region of Arkansas were collected and genotyped. Three of these accessions (ARK5-10, ARK5-11, and ARK5-13) were collected at relatively high elevations in the Ozarks (>425 m) after being identified as vigorous and cold hardy. The remaining sample (ARK5-12) was collected on UA Fruit Research Station (FRS) property approximately 400 m from the muscadine vineyard used for the UA breeding program. These four vines were included in the ARK5 population for all analyses requiring an individual be assigned to a population. Despite ranging from 20 to 65 km away from the ARK5 collection site, all individuals were collected from the Ozark Plateau Another accession collected in Arkansas (ARK2-11) was included with the closest wild population (ARK2) despite ARK2-11 being 50 km from the ARK2 collection site because both sampling locations were located in the Ouachita Mountains.

**Table 1:** Population names, number of individuals per population, locational data for each population, and summary statistics for basic population genetics parameters.

Pop. ID	Pop. description	*N*^z^	Latitude	Longitude	Elevation (m)	No. of private alleles	*H*_O_^y^	*H*_E_^x^	Alleles/locus
ARK	Arkansas selections	14	n/a	n/a	n/a	15	0.34	0.33	2.41
REC	Recent cultivars	18	n/a	n/a	n/a	2^w^	0.36	0.38	2.65
HIS^w^	Historical cultivars	24	n/a	n/a	n/a	21	0.38	0.40	3.16
VAR	Wild varieties	9	n/a	n/a	n/a	7	0.39	0.43	2.82
MUS^v^	*Muscadinia*	7	n/a	n/a	n/a	163	0.30	0.48	2.90
FLA	Anastasia, FL	11	29.8701	−81.2765	2	214	0.35	0.46	3.13
FLA2^u^	Nocatee, FL	n/a	30.0753	−81.3892	5	n/a	n/a	n/a	n/a
NC1	Ft. Macon, NC	12	34.6956	−76.6985	2	120	0.34	0.40	2.79
NC2	Umstead, NC	8	35.8702	−78.7468	130	24	0.34	0.43	2.83
NC3	Birkhead Mountain, NC	4	35.6370	−79.9040	192	6	0.32	0.41	2.01
MIS	Kiln, MS	11	30.4083	−89.4363	8	193	0.34	0.47	3.05
ALA	Blevin’s Gap, AL	9	34.6741	−86.5293	239	31	0.32	0.37	2.47
TEN	Harry Carter Area, TN	11	35.1224	−85.9200	386	16	0.32	0.38	2.63
OKL	Broken Bow, OK	7	34.1287	−94.6866	206	16	0.30	0.35	2.18
TEX	Indian Mounds, TX	3	31.3118	−93.6968	59	4	0.30	0.37	1.73
ARK1	Village Creek, AR	10	35.1557	−90.7206	100	9	0.31	0.39	2.68
ARK2	Pellegrino, AR	9	34.5180	−93.0002	311	13	0.34	0.37	2.57
ARK3	‘Y’ City, AR	4	34.7304	−94.0688	255	3	0.27	0.35	2.57
ARK4	Wildcat Mountain, AR	10	35.2768	−93.8050	208	30	0.28	0.32	2.21
ARK5	Jack Creek, AR	13	35.7085	−94.0960	286	16^t^	0.26	0.33	2.17

z Number of accessions.

y Observed heterozygosity.

x Expected heterozygosity.

w Three accessions (“Oh My!,” “Stuckey,” and “Spalding”) were not used to calculate private alleles. “Oh My!” is a hybrid with *Vitis vinifera* and was excluded from the recent cultivar summary statistics calculations. Both “Stuckey” and “Spalding” do not match their reported pedigrees and therefore were excluded from the historical cultivars.

v Muscadinia is composed of multiple *Vitis munsoniana* accessions, one *Vitis popenoei* accession, and one interspecific hybrid between all three subgenus Muscadinia species.

u The population FLA2 had only four accessions after data filtration (FLA-11, FLA-12, FLA-13, and FLA-14) and was combined with the FLA population that is only 25.1 km away.

t The accession ARK5-3 was excluded from summary statistic calculations due to a probable plating error.

### Cultivated Materials

In order to quantify the genetic diversity of cultivated muscadines as well as compare between cultivated and wild populations, 72 additional muscadine accessions were included in this study (Table 1; Supplementary Table 1). These vines represented wild selections that played a role in early crop improvement efforts, accessions maintained by germplasm repositories for diversity purposes, historical cultivars, recently released cultivars (REC), and advanced selections from the UA breeding program. Sources for this germplasm included the NCGR at Davis, CA, UGA, UA, and a private collection maintained by Gardens Alive! Inc. muscadine breeder Jeff Bloodworth. Accessions were assigned to five groups: *Muscadinia* varieties and accessions (MUS, *n* = 7), wild *V. rotundifolia* varieties (VAR, *n* = 9), historical cultivars released prior to 1970 (HIS, *n* = 24), recent cultivars released since 1970 (REC, *n* = 18), and unreleased selections from the UA muscadine breeding program (ARK, *n* = 14). Varieties were defined as cultivated vines selected from the wild, while cultivars were defined as cultivated vines resulting from controlled crosses in formal breeding programs. The *Muscadinia* (MUS) population was composed of wild *V. munsoniana* accessions (DVIT 2242, DVIT 2248, “Thornhill,” “Barrett Mtn,” and “Marsh”), one accession with *V. popenoei*, *V. munsoniana*, and *V. rotundifolia* parentage (“Fennel’s 3-way hybrid”), and the single *V. popenoei* (DVIT 2970) accession maintained by the NCGR. The 14 individuals within the Arkansas selections (ARK) population are advanced, unreleased genotypes from the UA breeding program. Each of these selections is preceded by the code “AM” (Arkansas Muscadine).

### DNA Extraction and Genotyping

Young leaf tissue was collected from wild muscadines for DNA extraction and genotyping and immediately placed in labeled 2 ml centrifuge tubes. Locational data for wild populations were recorded using OnX mapping software (OnX Maps, Missoula, MT, United States). Observational data, such as plant health, flower sex, and growing environment were noted at the time of collection when possible. Fresh tissue from the 72 additional accessions was sent to UA for extraction. DNA extraction followed a modified CTAB protocol (Porebski et al., 1997). The quantity of double-stranded DNA was verified using a Qubit Fluorometer (Thermo Fisher Scientific, Waltham, MA, United States). Final solution volume was diluted to 60 μl and stored at −80°C.

Genotyping was performed using RNase H2 enzyme-dependent amplicon sequencing (rhAmpSeq), a method that specifically targets amplicons within the genome (Dobosy et al., 2011). The accessions were genotyped with 2,000 rhAmpSeq haplotype markers distributed across all chromosomes with an average distance of 200 kb between each marker and specific emphasis in gene-rich regions. Illumina sequencing of rhAmpSeq amplicons allows for increased multiplexing capacity compared to AmpSeq (Yang et al., 2016) and captures haplotype alleles comprised of multiple SNPs and/or Indels per amplicon, resulting in highly informative markers (Zou et al., 2020). Marker development was accomplished using two publicly available *V. vinifera* genomes and *de novo* assemblies of seven *Euvitis* genomes, including wild American species such as *Vitis cinerea* and *Vitis rupestris*, although *V. rotundifolia* was not included in the development process (Zou et al., 2020). The 2,000 rhAmpSeq *Vitis* core markers were designed to target the “core genome” that was present in all nine grape genome assemblies in order to maximize transferability across species within subgenus *Euvitis*. Haplotype allele calls were generated using a pipeline designed to analyze the multiplexed amplicon sequencing data,^1^ resulting in a matrix composed of genotypes and markers with both alleles and read depth.

Data filtration was accomplished using RStudio and the package *adegenet*. A minimum read depth threshold of five reads per allele call was used. Monomorphic loci and loci with >20% missing data were removed. Loci putatively under selection were searched for using the R package dartR’s implementation of OutFLANK (Whitlock and Lotterhos, 2015). Population structure was analyzed using *structure* 2.3 software (Pritchard et al., 2000). The number of hypothesized populations (*K*) was run from 2 to 10 without *a priori* assumptions of population delineations and an admixture model. A burn-in period of 300,000 iterations was followed by 800,000 Markov-Chain Monte Carlo (MCMC) repetitions. The simulation was run three times for each *K* value. The most likely number of populations present in the data was determined using the delta *K* statistic (Evanno et al., 2005). Visualizations of the *structure* output were constructed using the *pophelper* package. An Analysis of molecular variance (AMOVA) was performed following Excoffier et al. (1992) in the R package *poppr* to investigate genetic variation based on the *a priori* assumption that each collection site sampled represented a distinct population. A dendrogram was created using an unweighted pair group method with arithmetic mean (UPGMA) tree based on Nei’s genetic distance using the packages *poppr* and *ape* (Nei, 1978). For each population, observed heterozygosity, expected heterozygosity, average number of alleles per locus, and the number of private alleles (alleles found in only one population) were calculated using the package *hierfstat*. Principal component analysis (PCA) was conducted using the package *ade4*. The package *ade4* was also used to conduct a Mantel test of the *de novo* wild populations using a genetic distance matrix calculated using *hierfstat* and a physical distance matrix calculated using *geosphere* (Mantel, 1967).

## Results

In this study, 152 wild *V. rotundifolia* accessions collected *de novo* and 72 additional wild and cultivated *Muscadinia* accessions were genotyped. Of the 2,000 markers, 1,276 had little to no amplification (defined as >80% missing data) and 37 loci were not polymorphic in this dataset. After filtering loci for missing data, 30 of the 152 wild accessions failed to amplify at >90% of loci and were removed. The failed reactions disproportionately affected the final sizes of the ARK3 (*n* = 4), TEX (*n* = 3), and NC3 (*n* = 4) populations (Table 1). Approximately 34.4% of the loci from the rhAmpSeq genotyping panel were polymorphic and had <20% missing data. The number of haplotype alleles per locus ranged from 2 to 24 with an average of 5.54. None of the 687 polymorphic haplotype loci were flagged as *F*_st_ outliers based on the null *F*_st_ distribution generated from OutFLANK (Supplementary Figure 1). To ensure that no loci potentially under selection were affecting analyses of population structure, *structure* analysis and the Mantel test were repeated with the full set of 687 polymorphic loci and with the 25 markers with the highest *F*_st_ excluded. There were no appreciable differences between the results of either analysis with the full and reduced datasets (data not shown). Therefore, the final dataset was composed of 194 unique genotypes and all 687 polymorphic haplotype loci.

### Heterozygosity, Private Alleles, and Alleles Per Locus

Observed heterozygosity ranged from 0.26 to 0.39 across the 15 wild and five cultivated populations (Table 1). The ARK5 and ARK3 populations, located in western Arkansas, had the lowest observed heterozygosity and the wild varieties (VAR) had the highest. Only the Arkansas selections (ARK) population had an observed heterozygosity higher than expected. The average number of alleles per locus for each population ranged from 1.73 to 3.16 (Table 1). TEX had the lowest number of alleles per locus as well as the lowest number of overall accessions. Only two wild populations (FLA and MIS) and one cultivated population (HIS) had an average of more than three alleles per locus. The Arkansas selections (ARK) had the lowest alleles/locus of the cultivated populations (2.41). The number of private alleles per population ranged from 2 to 214, with an average of 45. The wild varieties (VAR), Arkansas selections (ARK), historical cultivars (HIS), and recent cultivars (REC) all had lower than average number of private alleles despite having a higher than average number of individuals in each population (Table 1). The seven accessions in MUS had 163 private alleles. The three wild populations with the highest number of private alleles were FLA (214), MIS (193), and NC1 (120). TEX, ARK3, and NC3 each had five or fewer private alleles but also fewer than five individuals each (Table 1). Four individuals (ARK5-3, “Spalding,” “Stuckey,” and “Oh My!”) were excluded from calculations of private alleles and alleles per locus in their respective populations. The accession ARK5-3 appeared to be admixed with *V. munsoniana*, which was likely the result of an error during laboratory preparation of samples for genotyping. “Spalding” and “Stuckey” were excluded because initial results showed that they grouped with *V. munsoniana* despite there being no indication of *V. munsoniana* in their reported pedigrees. The cultivar “Oh My!” was developed from a seedless *V. vinifera* cultivar crossed with *V. rotundifolia* that was then backcrossed to *V. rotundifolia* multiple times, resulting in a cultivar with 23.1% of its genetic makeup from *V. vinifera* (Bloodworth, 2019). Due to its interspecific background, “Oh My!” inflated the number of private alleles in the recent cultivars (REC) population from 2 to 81 when it was included.

### Population Structure

**Figure 1:**
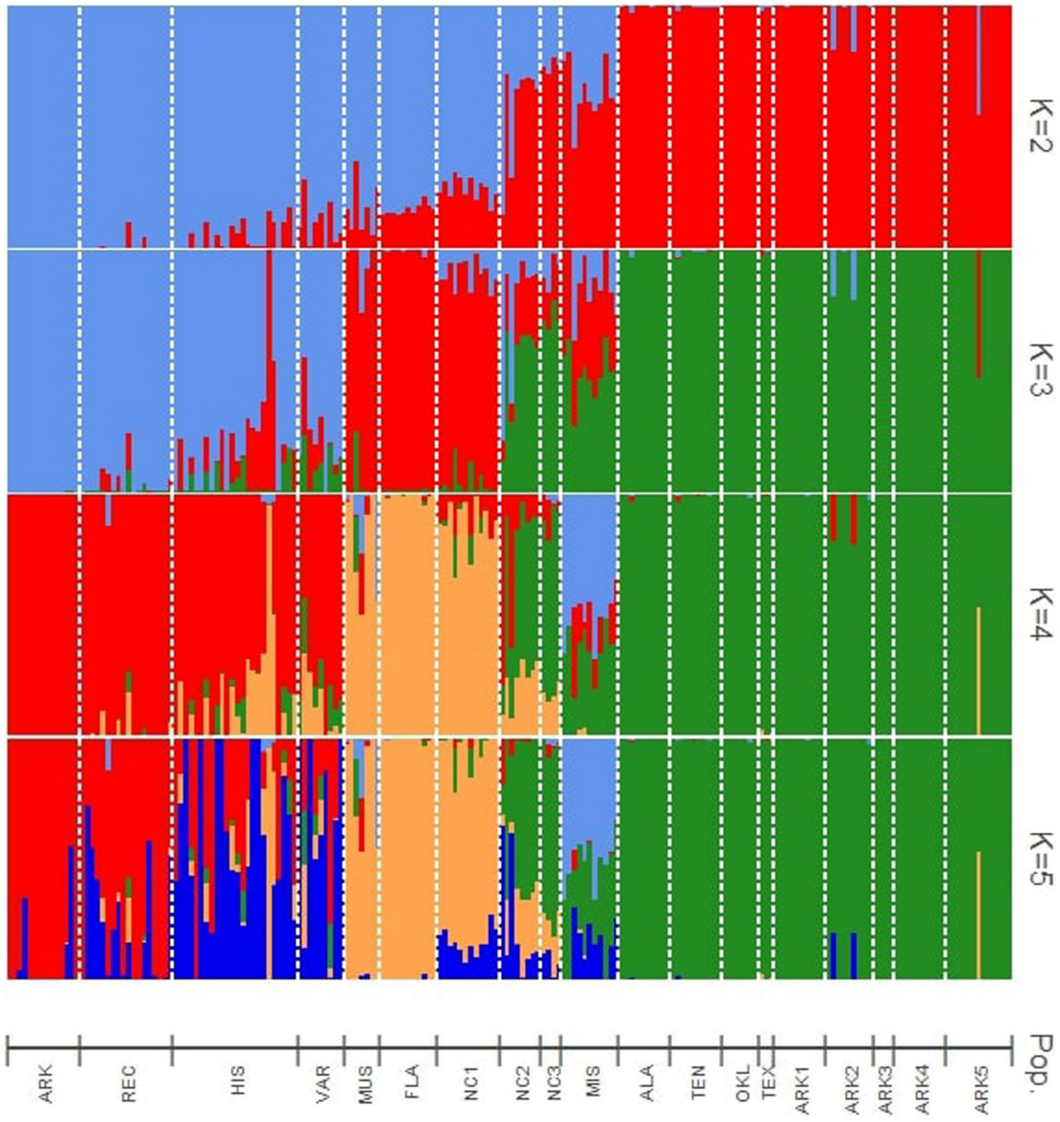
Bayesian structure analysis results showing assignments to four theoretical levels of *K* (2–5). Vertical bars indicate the estimated membership coefficients (Q) of each individual for each population cluster.

Four levels of *K* (2–5) are presented in this research. The statistic of Evanno et al. (2005) showed the highest probability for *K* = 3 (Figure 1). The hybrid nature of some wild populations warranted further investigation into population structure beyond *K* = 3 to better understand the genetic background of these populations. Cultivated populations also showed noteworthy admixture patterns at *K* values higher than 3. The results when the number of theoretical populations assumed in the *structure* analysis was greater than five were uninformative.

At *K* = 2, the cultivated materials (ARK, REC, HIS, and VAR) and *Muscadinia* (MUS) populations formed the first cluster, while the wild populations from west of the Appalachian Mountains (ALA, ARK1, ARK2, ARK3, ARK4, ARK5, OKL, TEN, and TEX) formed the second. Five populations (MIS, NC1, NC2, NC3, and FLA) collected within 250 km of the Atlantic or Gulf coasts showed moderate levels of hybridization between the two clusters. Each of the vines from the NC1 and FLA populations consistently had approximately 20% assignment to the second cluster, while the admixture levels in the MIS and NC2 populations were more variable from individual to individual.

**Figure 2:**
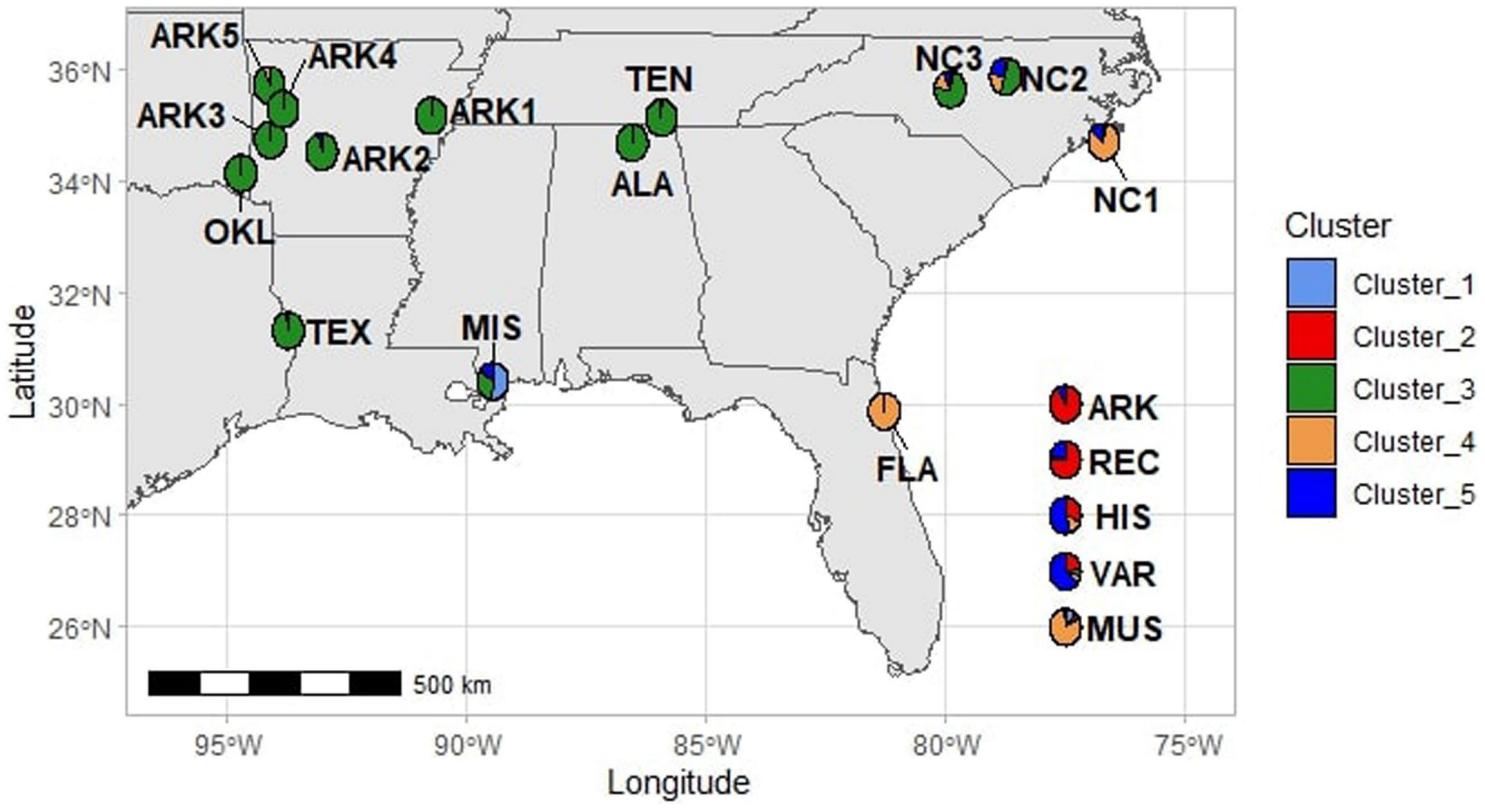
Map showing the collection sites of the wild populations used in this study as well as corresponding pie charts showing the average assignment to each cluster for each population at *K* = 5.

At *K* = 3, the cultivated populations generally grouped as one cluster with some notable exceptions. The historical cultivars “Spalding” and “Stuckey” had over 90 and 50% assignment, respectively, to the same cluster as MUS. The ARK population showed no levels of admixture with any other cluster while the other cultivated populations showed varying levels of admixture within each accession. The FLA, MUS, and NC1 populations were assigned to the second cluster with low to moderate levels of admixture with the other two clusters. Populations from Alabama, Arkansas, Oklahoma, Texas, and Tennessee (ALA, ARK1, ARK2, ARK3, ARK4, ARK5, OKL, TEN, and TEX), generally from the temperate regions of the southeastern U.S., formed a third cluster with low levels of admixture compared to other populations. The KIL, NC2, and NC3 populations appeared to be mixtures of all three hypothetical populations assumed for *structure* simulation. Despite being geographically equidistant from the populations collected in North Carolina and the populations collected in Arkansas, the ALA and TEN populations showed no admixture with material from North Carolina and grouped exclusively with material from Arkansas, Oklahoma, and Texas (Figure 2).

At *K* = 4, the MIS population became a distinct cluster that was only observed at minor frequencies in other populations. However, MIS continued to show admixture from the remaining clusters. Each individual within MIS had low to moderate levels of admixture with the cluster of Arkansas, Alabama, Oklahoma, Tennessee, and Texas populations. The *K* = 5 *structure* simulation split the cultivated populations into two groups. In the VAR population, only the cultivar “Thomas” had a majority assignment the old second cluster and all other accessions in VAR had majority assignment to the new fifth cluster. For the HIS population, six cultivars were majority assigned to second cluster and 11 to the new fifth cluster. In REC, 14 of the 18 cultivars were assigned to cluster 2. Only one accession in the ARK population, AM-77, had majority assignment to the new fifth cluster (Figure 2).

**Figure 3:**
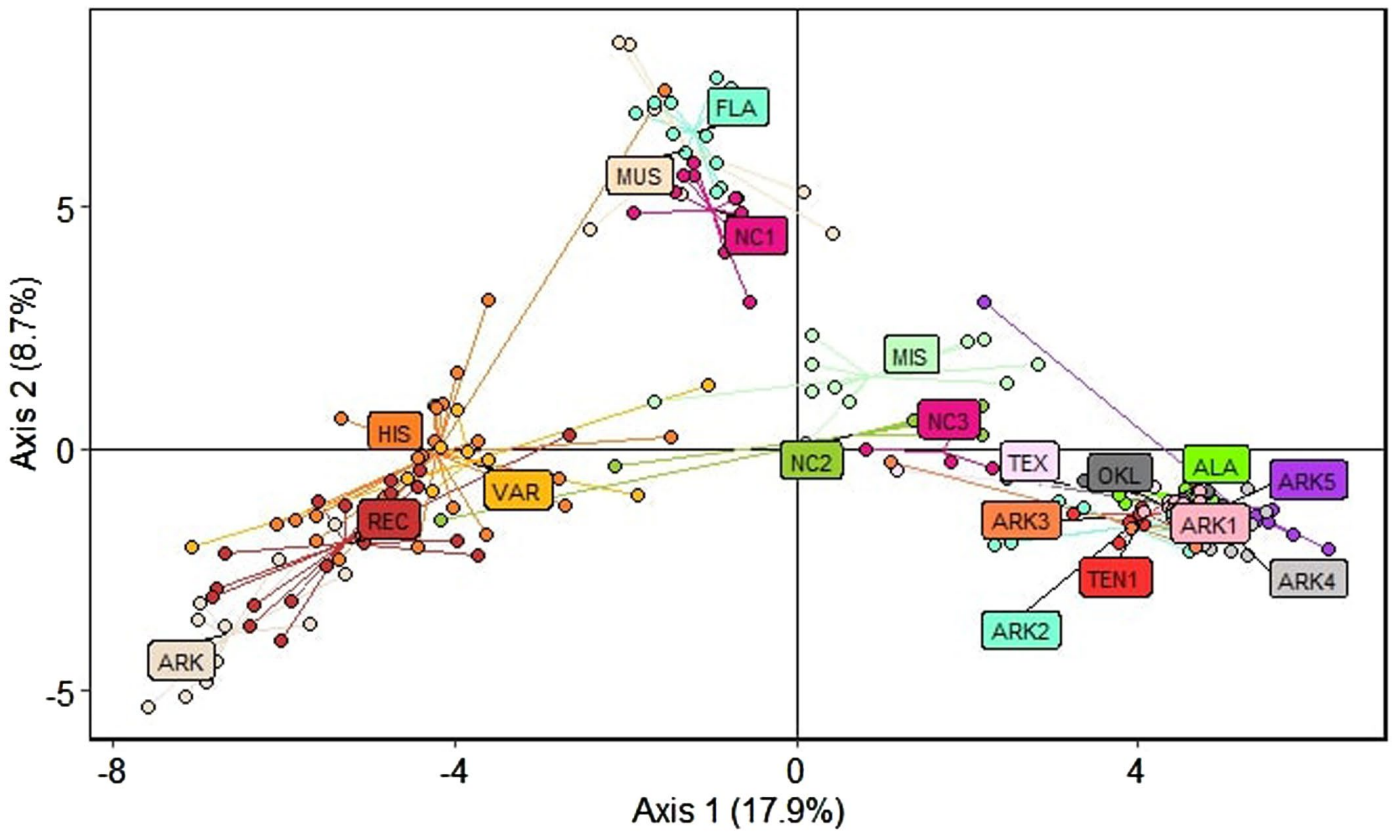
Principal component analysis (PCA) showing the three distinct clusters of material used in this study. Each point is color coded according to its respective population.

Principal component analysis (PCA) produced three clusters corresponding to the *structure* results at *K* = 3 (Figure 3). The first axis accounted for 17.9% of variation, with cultivated material to the left of the axis and wild material on the right side of the axis. A second axis accounting for 8.7% of observed variation separated the subgenus *Muscadinia* (MUS), Florida (FLA), and coastal North Carolina (NC1) populations from the cultivated populations and the wild populations collected in Arkansas, Alabama, Oklahoma, Tennessee, and Texas. Three populations (NC2, NC3, and MIS) that showed significant admixture in the *structure* analysis grouped closest to the wild populations but were distinct from the tightly-grouped cluster of populations from Arkansas, Alabama, Oklahoma, Tennessee, and Texas.

**Figure 4:**
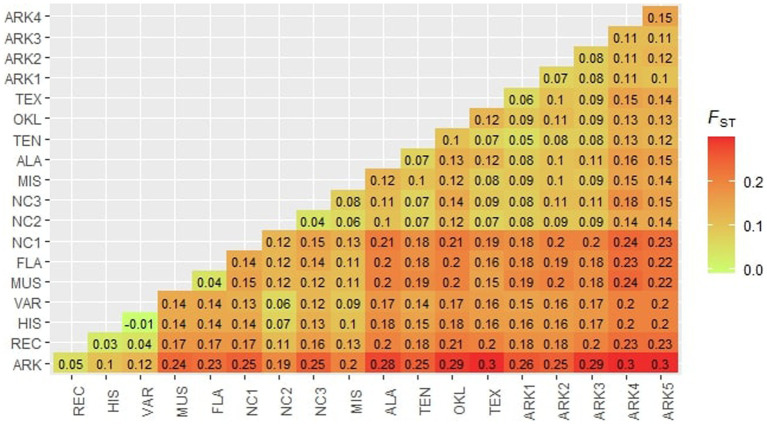
Pairwise *F*_ST_ values calculated for each population following Weir and Cockerham (1984).

Pairwise *F*_st_ values ranged from −0.01 to 0.31, indicating that there was significant structure among the populations in this study (Figure 4). The highest observed F_st_ values were between the ARK population and the Arkansas Ozarks populations ARK5 and ARK4 (0.30 and 0.31, respectively). The NC1 population had pairwise *F*_st_ values greater than 0.20 for most wild populations collected west of the Appalachian Mountains. Both FLA and MUS had pairwise *F*_st_ values ranging between 0.11 and 0.24 when compared to the 18 other populations used in this study. The populations collected in Alabama, Arkansas, Oklahoma, Tennessee, and Texas were generally undifferentiated from one another, the majority of comparisons having *F*_st_ values of less than 0.15. The cultivated populations in this study also showed relatively little differentiation. Historical cultivars (HIS) and wild varieties (VAR) had the lowest observed *F*_st_ of all the comparisons of −0.01. Pairwise *F*_st_ values for the ARK compared to other cultivated populations decreased from the wild varieties (VAR; 0.12) to historical cultivars (HIS; 0.11) to recent cultivars (REC; 0.05). AMOVA results supported the high *F*_st_ values between populations, showing that just over 14.5% of genetic variation was among populations, 4.7% of variation was within populations, and 80.8% of variation was within individuals. The Mantel test showed a significant positive association between genetic distance and physical distance (*r* = 0.47, *p* = 0.002).

**Figure 5:**
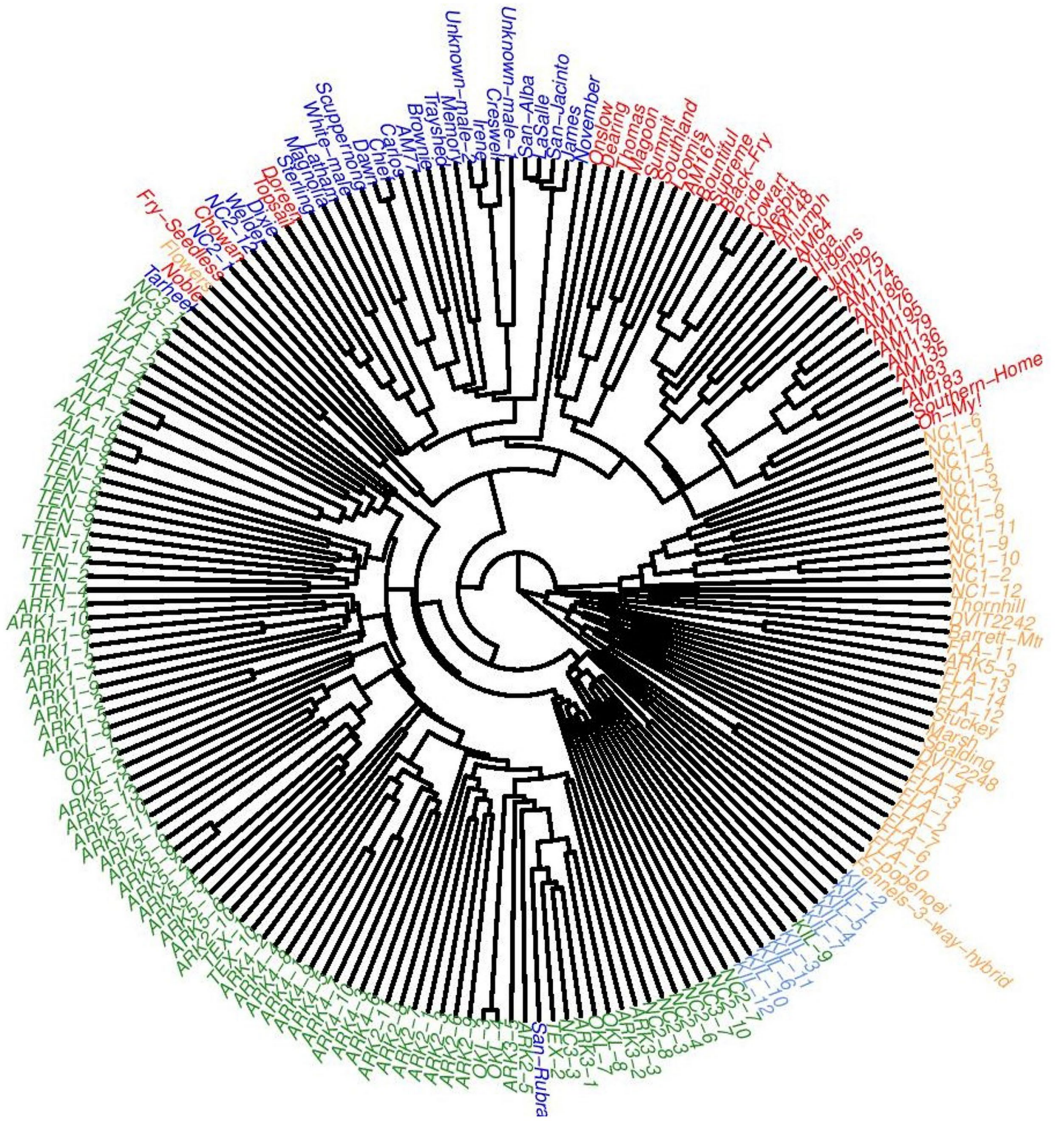
Unweighted pair group method with arithmetic mean (UPGMA) tree of all 194 accessions used in this study colored according to which cluster they were majority assigned to at *K* = 5.

An UPGMA tree was constructed using genetic distances calculated following Nei (1978) that further detailed the population structure of this dataset (Figure 5). One clade was formed by two accessions: the *V. popenoei* sample (DVIT 2970) and “Fennell’s 3-way Hybrid,” which is one half *V. popenoei*, one quarter *V. munsoniana*, and one quarter *V. rotundifolia* by pedigree. The FLA population grouped most closely with the *V. munsoniana* material included in this study. The NC1 population, consistent with both the *structure* and PCA results, was a separate clade despite being closely related to the MUS and FLA material. Cultivated and wild materials generally formed separate clades. Only one cultivated accession (“San Rubra”) grouped with wild material. Two genotypes from the NC2 population (NC2-1 and NC2-12) grouped within the cultivated material.

## Discussion

This research represents the most extensive *V. rotundifolia* genetic diversity study conducted to date. One hundred and ninety-four individuals and 687 loci were used to evaluate population structure and quantify genetic diversity of wild and cultivated muscadine populations. Prior genetic diversity work in muscadines focused mainly on cultivated accessions or the relationship between muscadines and *Euvitis* grapes (Qu et al., 1996; Riaz et al., 2008; Cao et al., 2020). One previous study used nine wild accessions available through the NCGR at Davis, CA, but did not incorporate wild genotypes collected *de novo* (Cao et al., 2020). Other research has sampled from multiple wild populations, but the geographic focus of that study was limited to North Carolina, South Carolina, and Florida (Smith, 2010). The results described in this paper represent the first instance wild populations from the western range of muscadines have been sampled for genetic characterization. Additionally, 65 cultivated accessions and seven germplasm accessions previously characterized as *V. munsoniana*, *V. popenoei*, and other subgenus *Muscadinia* interspecific hybrids were included in the study. Wild varieties, early improved genotypes, cultivars released since 1970, and advanced breeding selections were included in this analysis.

### Effectiveness of rhAmpSeq in Muscadine Grapes

The rhAmpSeq *Vitis* core panel of haplotype markers used in this study was developed to have marker transferability >90% between species within subgenus *Euvitis* that diverged up to 20 mya (Zou et al., 2020). However, the three species in subgenus *Muscadinia* likely diverged from subgenus *Euvitis* species approximately 37 mya (Liu et al., 2016), and none of these species were included in the design and optimization of the *Vitis* core markers. Still, major synteny between muscadine and bunch grape genomes has been found despite the significant time since divergence and difference in chromosome number (Lewter et al., 2019; Cochetel et al., 2021; Park et al., 2022). In this study, we found 34.4% of rhAmpSeq markers developed for *Euvitis* grapes were transferable to diverse *Muscadinia* germplasm. One previous study using the *Vitis* rhAmpSeq markers for a GWAS analysis in a muscadine breeding population was able to utilize 1,283 markers (64.2% transferability) after a data imputation process (Park et al., 2022). The average read depth per sample obtained by Park et al. (2022) was similar to this study. Therefore, the lower number of transferable *Vitis* rhAmpSeq markers useful markers discovered in this study can likely be attributed to fact that we required a minimum read depth of five for allele calling and did not attempt imputation for missing genotype data.

### Comparisons to Previous Genetic Studies

Multiple differences were found between the results of this study and past muscadine genetic diversity studies. The exceptionally high genetic diversity observed in a small set of wild muscadine germplasm accessions by Cao et al. (2020) was not observed in all populations collected for this study. The majority of the western wild populations collected for this study had relatively few private alleles. The five populations collected in Arkansas, despite being collected from diverse climatic and ecological regions of the state, contained 3–35 private alleles each. These values represent less than 20% of the number of private alleles found in MIS (193) or FLA (214). The MIS and FLA populations each had more private alleles and a higher average number of alleles per locus than the MUS population, which consisted of seven individuals from interspecific backgrounds: one *V. popenoei* accession, two wild *V. munsoniana* accessions, three *V. munsoniana* varieties, and the interspecific accession “Fennel’s 3-way hybrid.” Directly comparing the number of private alleles found in this study to Cao et al. (2020) is difficult. The latter used 20 SSR markers specifically selected for high differentiation in muscadines, while this study used 687 haplotype markers designed for amplification and polymorphism in diverse *Euvitis* material. Another reason for the high observed diversity the nine wild accessions from Cao et al. (2020) is that this group represented all three species in subgenus *Muscadinia* and was sampled from across the native range of *V. rotundifolia* using accessions available through the USDA germplasm repository and a private collection, including one accession each from Arkansas and Louisiana.

Heterozygosity of the wild material collected in this research was generally lower than previously reported in other studies. Every wild population sampled for this study had observed heterozygosity lower than expected heterozygosity. Smith (2010) sampled 24 wild populations and calculated observed heterozygosity levels ranging from 0.27 to 0.65. Cao et al. (2020) found an observed heterozygosity level of 0.71 for the set of wild accessions used in that study. However, the observed heterozygosity was lower than the expected heterozygosity, as we found in this study. In this study, observed heterozygosity levels ranged from 0.27 to 0.35 in the 15 wild populations. As with the calculations alleles per locus, the type of markers used may have impacted our results. Smith (2010) and Cao et al. (2020) each used 15–20 SSR markers selected for their high levels of polymorphism in muscadines. When the dataset used in this study was adjusted to include only the 30 loci with the highest number of alleles, the range of observed heterozygosity levels increased from 0.27–0.39 to 0.50–0.75 across the 20 populations. The population with the lowest observed heterozygosity in this study (ARK5) is located at the northernmost extent of the native range of muscadines in the Arkansas Ozark Plateau. Other populations collected near the westernmost extent of the native range (ARK3, ARK4, OKL, and TEX) also had lower observed heterozygosity compared to the wild populations collected in eastern and central Arkansas, Alabama, Florida, Mississippi, North Carolina, and Tennessee.

The cultivated material used in this study also had lower observed heterozygosity than other examples in the literature. Riaz et al. (2008) evaluated 57 cultivated genotypes with 15 SSR loci and found an average observed heterozygosity of 0.76, which was higher than the expected heterozygosity of 0.69. Cao et al. (2020) found a similar trend of cultivated material having higher heterozygosity than expected for both historical and current cultivars. In this study, only the Arkansas selections (ARK) had higher observed than expected heterozygosity, although ARK had also had the lowest observed heterozygosity of the four cultivated populations. Observed heterozygosity levels decreased gradually from the wild varieties (VAR), to the historical cultivars (HIS), recent cultivars (REC), and Arkansas selections (ARK), potentially indicating a loss in genetic diversity over time across breeding germplasm. A similar trend is observed with the number of alleles per locus, although HIS is a notable exception (Table 1). One reason for this could be that HIS is by far the largest population examined in this study with 24 accessions. The number of private alleles is low for each cultivated population relative to many of the wild populations, particularly when accounting for the larger sample sizes of the cultivated populations. This is not unexpected, particularly in the case of the Arkansas Selections (ARK) as many of the most important cultivars used as parents in the founding of the program that could have been the source of unique alleles have also been included in the study.

### Population Structure of Subgenus *Muscadinia* and Wild Populations

The taxonomic distribution of muscadines and the other 2*n* = 40 *Muscadinia* species is a matter of some discussion, made more difficult by the overlapping ranges that these species inhabit (Olien, 1990; Aradhya et al., 2013). *Vitis munsoniana* is at times considered a subspecies of *Vitis rotundifolia* that is simply better adapted to semitropical environments (Olien, 2001), although T.V. Munson felt the differences between *V. rotundifolia* and *V. munsoniana* were clearer than many of the differences between American *Euvitis* species (Munson, 1909). *Vitis munsoniana* is native to Florida and the Gulf Coast and has not been reported in the interior of the Southeast (Husmann and Dearing, 1913). Two populations (MIS and FLA) were collected within the documented geographic range of both *V. rotundifolia* and *V. munsoniana* (Munson, 1909; Dearing, 1947). At *K* = 2 and *K* = 3, the MIS population appeared to be the result of hybridization between western *V. rotundifolia* populations and coastal *V. munsoniana* populations. At *K* = 4 and *K* = 5, it appeared that some of this admixture was from a unique population, perhaps a coastal *V. rotundifolia* population, that only appeared sparingly in other accessions in this study. The MIS samples also clustered between the three major groups of wild and cultivated material in the PCA. At all levels of *K*, the MIS population showed some level of admixture with cultivated populations. Approximately 13 km south of the MIS collection site are the remains of Brown’s Vineyard, a historical muscadine vineyard that grew “Scuppernong” on at least 15 acres for decades (Stafne, 2014). It is possible that historical admixture between local wild vines and the vineyard have resulted in the unique genetic profile observed in the MIS population.

Results from the PCA, *structure* analysis, and pairwise *F*_st_ comparisons showed that FLA was highly related to MUS, indicating that the wild population was likely almost entirely *V. munsoniana* in origin (Table 1; Figures 1 4). One unexpected result of this study was the close relationship between the NC1, MUS, and FLA populations (Figures 1–5). The NC1 population was sampled from a maritime forest in Ft. Macon State Park on the southern Outer Banks of the Atlantic coast of North Carolina, well outside the reported native range of *V. munsoniana*. However, this population appeared much more closely related to *V. munsoniania* than the other muscadine germplasm included in this study, including other populations sampled from the North Carolina Piedmont (NC2 and NC3) and historically important cultivated varieties (VAR) that were largely sourced from the North Carolina Coastal Plain. In contrast, Smith (2010) found that the wild populations from Florida were genetically distinct from those of North Carolina, although three of the 14 North Carolina populations from that study were sampled from sites on the Outer Banks and the FLA population in this study was sampled from the same location as one of Smith’s eight Florida populations. The NC1 population is located in USDA Plant Hardiness Zone 8a and experiences colder temperatures than the semi-tropical regions of the Gulf and Atlantic coasts that traditionally have been considered the historical range of *V. munsoniana*. Munson (1909) noted that *V. munsoniana* was almost as cold hardy as *V. rotundifolia* and had fruited for multiple years his vineyard in Denison, Texas (also Zone 8a). Thus, it seems possible that the far southern Outer Banks could be a sort of isolated refugium for *V. munsoniana* or an underexplored intermediate bridge between the two species or subspecies. This hypothesis should be explored further by sampling coastal populations in South Carolina and Georgia.

### Diversity of Wild Populations From Alabama, Arkansas, Oklahoma, Tennessee, and Texas

The wild populations from Alabama, Arkansas, Oklahoma, Tennessee, and Texas in this study were more genetically similar than expected. *Vitis rotundifolia* has a large native range, and it was hypothesized that genetic diversity within the species would be evenly distributed across the southeastern U.S. The *structure* and PCA results showed that even populations such as ALA and ARK3 that were collected more than 800 km apart were genetically similar (Figures 2, 3), although a Mantel test did find that increased physical distance was positively associated with genetic distance. It is not immediately clear why these western interior populations are not more genetically differentiated. Results from this study show that coastal areas are major regions of diversity for subgenus *Muscadinia*. It is possible that the regions sampled for this study have had shifts in climate to make them more suitable to muscadines since the last glacial maximum during the ice age approximately 10,000 years ago.

One notable exception to the lack of differentiation in western populations was the accession ARK5-3, which appeared to be admixed between the western population cluster and the subgenus *Muscadinia* cluster. However, these results are likely due to a laboratory plating error, as there is no evidence to support that there are *V. munsoniana* × *V. rotundifolia* hybrids occurring naturally in the Arkansas Ozarks. Two accessions in the ARK2 population showed low levels of admixture with cultivated populations. A possible explanation for this finding is that a small muscadine vineyard had recently been planted on the property where the ARK2 population was sampled, and some of the wild vines used in this study were young enough to be the result of hybridization between wild and cultivated material. In contrast, one vine sampled on the UA Fruit Research Station property (ARK5-12) showed no admixture with cultivated populations despite its proximity to the muscadine vineyard used by the UA breeding program. Admixture between cultivated and wild populations has been shown to decrease the unique genetic diversity of wild populations in a process known as genetic swamping. This phenomenon has been observed in *Vitis* previously, such as regular hybridization between *V. californica* Benth. and *V. vinifera* leading to genetic diversity being lost in the former (Dangl et al., 2015). The small sample of wild vines growing near vineyards in Arkansas used in this study shows that while some level of admixture occurs between these populations, it does not appear that cultivated populations are swamping out local Arkansas wild populations of *V. rotundifolia*.

### Diversity of Central North Carolina Populations

The two populations collected in the Piedmont of North Carolina (NC2 and NC3) showed more admixtures in the *structure* and PCA analyses than many of the other wild populations sampled in this study. Most of the wild vines that were cultivated by growers in the 19th and early 20th centuries that were later used as founding parents in breeding programs were sourced from forests in the Coastal Plains of North Carolina, between the location of these two populations and the NC1 population. Both populations showed low levels of admixture with the MUS, FLA, and NC1 populations at all tested levels of *K* (Figure 1), likely a result of their proximity to the coastal NC1 population. The varying admixture levels with cultivated material observed in the NC2 population could be the result of feral vines that escaped cultivation. The two accessions from NC2 that showed significant admixture with cultivated material, NC2-1 and NC2-12, grouped most closely in the UPGMA dendrogram with the cultivar “Chowan,” a bronze-fruited cultivar released in the 1960s. Bronze fruit were observed on NC2-1, a trait that is exceedingly rare in wild muscadines and common in cultivated material. These findings suggest that NC2-1 and NC2-12 probably resulted from accidental cross pollination between cultivars grown at old homesteads and wild populations in the surrounding forest. Smith (2010) also observed the bronze phenotype in wild material from collections made around Raleigh, NC. Therefore, the long cultivation history of muscadines in North Carolina may have led to more genetic swamping in North Carolina than in Arkansas.

### Use of Wild Populations in Cultivar Development

The results of this study indicate that the wild muscadine populations from the western part of the native range are highly differentiated from the germplasm used in muscadine breeding during the last century. The PCA axis that accounts for the highest variation (17.9%) showed the wide gulf between the material collected in Alabama, Arkansas, Oklahoma, Tennessee, and Texas and both recent cultivar releases and the advanced breeding selections within the Arkansas muscadine breeding program (Figure 3). This finding is not surprising considering that the founding germplasm used in muscadine breeding programs was sourced from a very limited portion of their native range. The origins of nearly every muscadine variety in the early 20th century can be traced to wild vines collected in eastern North Carolina (Husmann and Dearing, 1913). Wild *Muscadinia* germplasm from Florida has also been important in muscadine breeding. The “H1” source of hermaphroditism in muscadines was selected from a cross between North Carolina varieties and wild Florida pollinizers and used extensively in subsequent breeding efforts (Dearing, 1917; Smith, 2010). The importance of wild Florida germplasm in historical breeding material can be observed in our results. “Tarheel,” an important historical cultivar and parent that is the closest existing relative of the “H1” source of hermaphroditism is predicted to be 12.5% *V. munsoniana* by pedigree. We found that “Tarheel” had 27% assignment to the *structure* cluster with the MUS, FLA, and NC1 populations at *K* = 5 and grouped closer to the *V. munsoniana* material than other cultivated material in the PCA.

Although the wild populations from the western portion of the native range of muscadines had lower genetic diversity than the coastal populations (FLA, MIS, and NC1) sampled in this study, the presence of unique alleles within each population indicates the potential for beneficial alleles in these populations. Cold tolerance is of particular importance to the UA muscadine breeding program because of its location near the northern limits of the native range of muscadines. Damage from winter temperatures was rarely observed on wild muscadine vines during tissue collection in this study. The ARK5 population was collected from a site further north and at higher elevation than the UA breeding program vineyard in Clarksville, AR. Vines at the ARK5 collection site were regularly observed to survive past juvenility, indicating that this was an established population adapted to local winter conditions. Crossing with wild muscadine vines from the southern Ozarks could help achieve a major breeding goal of the UA muscadine breeding program by serving as a source of cold hardiness. It would not be the first time that wild material was found to contain beneficial traits. Bronze-fruited muscadines are considered superior for winemaking due to a lack of diglucoside anthocyanins that cause unsightly browning during the aging process. Using HPLC, Goldy et al. (1989) characterized the anthocyanin content of black fruit from wild muscadines in Arkansas and North Carolina and found superior monoglucoside anthocyanin profiles for winemaking compared to current cultivated material.

### Differences Among Cultivated Material

Results from the PCA show a steady differentiation over time of cultivated material from the two clusters of wild populations (Figure 3). Historical cultivars (released prior to 1970) and wild varieties were relatively dispersed across the PCA plot compared to both wild and other cultivated populations. The dispersion of the historical cultivars is expected as the categorization of “historical cultivar” as defined in this study is quite broad compared to the other cultivated population categorizations. In this study, a historical cultivar could be the offspring of two wild varieties such as “San Jacinto” (and therefore relatively undifferentiated from wild types) or the result of multiple generations of controlled crossing such as “Chowan.”

The UA muscadine breeding program began in 2007 and has utilized recently released cultivars, such as “Tara,” “Supreme,” and “Southern Home” as a base for genetic improvement. “Southern Home” is an interspecific hybrid with *V. rotundifolia*, *V. popenoei*, and *Euvitis* material in its pedigree (Mortensen et al., 1994). It is therefore unsurprising that the advanced selections within the program represent the most distal cluster along both axes in the PCA plot. A small decrease in heterozygosity levels was observed along the progression from the VAR, HIS, REC, and ARK populations, representing an increase in inbreeding as crop improvement efforts transitioned from selecting vines in the wild to breeding efforts over 100 years later. The increasing levels of inbreeding in more recently released muscadine cultivars has also been documented by pedigree analysis (Williams et al., 2021). The low number of private alleles and the decrease in the average alleles per locus from historical cultivars to recent cultivars and the unreleased material in the UA breeding program also represent a challenge for breeders. Without new beneficial alleles, genetic gains will be more difficult to achieve. Interestingly, the number of private alleles in the REC population increased from 2 to 81 when “Oh My!” a *Euvitis* × *Muscadinia* hybrid that is predicted to be 23.1% *V. vinifera* by pedigree (Bloodworth, 2019). Therefore, crosses with wild germplasm from diverse parts of the native range and with diverse *Vitis* species are both options to increase genetic diversity in cultivated muscadine breeding material.

### Potential Misidentifications in Muscadine Germplasm

T.V. Munson produced a number of putative hybrids between “Scuppernong” and *Euvitis* grapes (Munson, 1909), yet the Munson cultivars included in this study (“San Jacinto,” “La Salle,” “San Alba,” and “San Rubra”) do not appear to be of *Euvitis* descent. Uncertainty about the provenance of these cultivars had been expressed by Dearing as early as 1917, and genetic research conducted with these varieties has provided further evidence (Dearing, 1917; Cao et al., 2020). In this study, “San Jacinto,” “La Salle,” and “San Alba” grouped closely together with the eastern North Carolina wild variety “James” in the UPGMA dendrogram and clustered with the other historical cultivars in the PCA plot. Other misidentifications observed in previous genetic studies were also confirmed by our research. Genetic and phenotypic inconsistencies reported for “Creswell,” “Irene,” “Stuckey,” and “Spalding” also appear in our dendrogram (Riaz et al., 2008; Cao et al., 2020). “Stuckey” and “Spalding” in this study appear to be *V. munsoniana* in origin despite their reported pedigrees being exclusively *V. rotundifolia* (Figures 3, 5). It is likely that these accessions are mislabeled in the NCGR germplasm collection.

## Conclusion

Although a Mantel test found that genetic distance between wild populations increased with physical distance, *structure* and principal component analyses showed that many of the muscadine populations collected west of the Appalachian Mountains were more genetically homogenous than expected. However, the same analyses confirmed that these populations have not been incorporated in either historical or present breeding efforts and could serve as a source of beneficial alleles for current breeding programs. Coastal populations of *V. rotundifolia* and *V. munsoniana* were the most diverse wild populations examined in this study. One of these populations, collected in the Outer Banks of North Carolina, appeared to be *V. munsoniana* in origin despite its location far north of that species’ previously documented range. The *structure* and principal component analyses also showed that the last century of muscadine breeding efforts have resulted in significant differentiation from the wild material that formed the foundation of early muscadine breeding programs. Calculations of heterozygosity, private alleles, and the average alleles per locus for these cultivated populations reinforced the findings of recent studies showing increased inbreeding coefficients and low numbers of unique alleles in recent muscadine cultivars. The 2,000 rhAmpSeq *Vitis* core panel markers, despite being developed for subgenus *Euvitis* material, had 34.4% transferability to the subgenus *Muscadinia* populations used in this study. The final dataset comprised of 194 muscadine accessions and 687 haplotype markers, representing the largest investigation of genetic within subgenus *Muscadinia* to date. Low observed heterozygosity levels, likely an artifact of marker design, did not prevent rhAmpSeq from being an effective genotyping platform for cultivar fingerprinting and establishing geospatial patterns of population structure in both cultivated and wild muscadine grapes.

## Data Availability Statement

The original contributions presented in the study are included in the article/Supplementary Material, further inquiries can be directed to the corresponding author.

## Author Contributions

KB conducted extractions and analyses and drafted the manuscript. MW provided overall conceptual guidance and funding. KB and MW participated in the collection of tissue from wild muscadine populations and edited and revised the manuscript collaboratively. All authors contributed to the article and approved the submitted version.

## Funding

The genotyping was funded by *Vitis*Gen2, the US Department of Agriculture (USDA)-National Institute of Food and Agriculture (NIFA) Specialty Crop Research Initiative, award No. 2017-51181-26829. Additional funding for this research came from Hatch Project ARK02599.

## Conflict of Interest

The authors declare that the research was conducted in the absence of any commercial or financial relationships that could be construed as a potential conflict of interest.

## Publisher’s Note

All claims expressed in this article are solely those of the authors and do not necessarily represent those of their affiliated organizations, or those of the publisher, the editors and the reviewers. Any product that may be evaluated in this article, or claim that may be made by its manufacturer, is not guaranteed or endorsed by the publisher.

## Footnotes

^1^ https://bitbucket.org/cornell_bioinformatics/amplicon

## Supplementary Material

Supplementary Figure 1 Results from OutFLANK analysis including **(A)** a scatterplot of expected heterozygosity and *F*_st_ showing that no alleles were flagged as outliers putatively under selection and **(B)** a histogram showing the distribution of alleles with varying *F*_st_ values.

Supplementary Table 1 Accession name, population, year of release (when applicable), and source of tissue for the 194 accessions used in this study. Included are the structure proportional assignments to each cluster at *K* = 5 and the coordinates of the first two principal components.
