# Supplementary figures and images for: Genetic Diversity of Wild and Cultivated Muscadine Grapes (Vitis rotundifolia Michx.)
Source: Front Plant Sci. 2022 Mar 28;13:852130. doi: 10.3389/fpls.2022.852130 (PMC8996184; doi:10.3389/fpls.2022.852130)

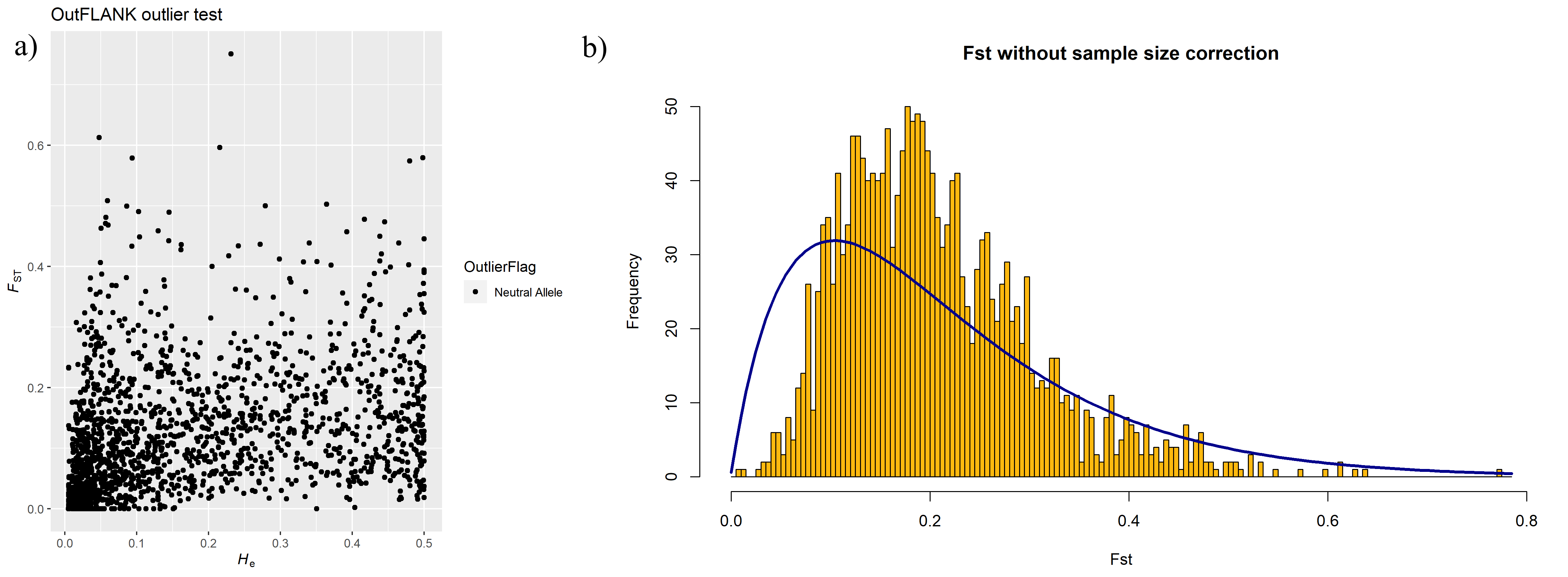

Supplement: Supplementary Figure 1 — Results from OutFLANK analysis including (A) a scatterplot of expected heterozygosity and Fst showing that no alleles were flagged as outliers putatively under selection and (B) a histogram showing the distribution of alleles with varying Fst values. [file Image_1.PNG]
